# Validation of an Aesthetic Assessment System for Commercial Tasks

**DOI:** 10.3390/e24010103

**Published:** 2022-01-09

**Authors:** Nereida Rodriguez-Fernandez, Sara Alvarez-Gonzalez, Iria Santos, Alvaro Torrente-Patiño, Adrian Carballal, Juan Romero

**Affiliations:** Department of Computer Science and Information Technologies, Faculty of Communication Science, University of A Coruña, 15071 A Coruña, Spain; nereida.rodriguezf@udc.es (N.R.-F.); sara.alvarezg@udc.es (S.A.-G.); iria.santos@udc.es (I.S.); alvaro.torrente@udc.es (A.T.-P.); adrian.carballal@udc.es (A.C.)

**Keywords:** aesthetics, e-commerce, real estate, validation, digital images

## Abstract

Automatic prediction of the aesthetic value of images has received increasing attention in recent years. This is due, on the one hand, to the potential impact that predicting the aesthetic value has on practical applications. Even so, it remains a difficult task given the subjectivity and complexity of the problem. An image aesthetics assessment system was developed in recent years by our research group. In this work, its potential to be applied in commercial tasks is tested. With this objective, a set of three portals and three real estate agencies in Spain were taken as case studies. Images of their websites were taken to build the experimental dataset and a validation method was developed to test their original order with another proposed one according to their aesthetic value. So, in this new order, the images that have the high aesthetic score by the AI system will occupy the first positions of the portal. Relevant results were obtained, with an average increase of 52.54% in the number of clicks on the ads, in the experiment with Real Estate portals. A statistical analysis prove that there is a significant difference in the number of clicks after selecting the images with the AI system.

## 1. Introduction

In a reality in which a large number of consumers are connected to the Internet, there are countless stimuli and options that they receive every day. On this basis, it is not surprising that more and more product choices are being made based on the aesthetic value and distinctiveness of visual design [1]. On the other side, companies are beginning to see the need to find new ways to be competitive, to attract and retain customers and to maintain or increase sales [2,3,4]. As Deng and Poole state, this requires a change in approach to design and user experience and a greater emphasis on aesthetics and the emotional responses it elicits. Through stimulated emotional responses, aesthetics can contribute to the success of e-commerce and encourage desirable user behaviours such as spending more time browsing, exploring more varied products, responding better to promotional initiatives, and increasing the likelihood of purchase [5]. In addition, after COVID-19, this adaptation must be even greater in the digital sphere, since the pandemic has contributed to the increase in the digitization of consumers [6]. In e-commerce, the products are perceived through their photographs and it is in them where consumers judge the aesthetic value discussed.

The great advances in technologies related to image capture and the growth of other fields such as visual computing [7,8] have given the average user more power and capacity in image acquisition. From the user’s point of view, these new technologies create the expectation of more attractive images, but this also requires the knowledge and execution of basic aesthetic principles during capture and editing [9]. In this context, having systems that provide automatic aesthetic feedback is of great practical relevance.

The purpose of the computational aesthetic evaluation is to simulate the visual system and human perception to make an aesthetic judgment about the images automatically [10]. In recent years, many researchers from different fields of knowledge, such as Artificial Intelligence, Psychology, Arts or Design, have focused on the identification of the characteristics most related to human aesthetic preferences, as well as on the modelling of computer systems to recreate human evaluations for classification and prediction tasks [7,11,12,13,14,15,16,17,18,19].

In recent years, our research group has developed a novel genetic search correlation model for aesthetic evaluation [20,21]. This model has been implemented in a system capable of predicting the aesthetic value of images belonging to the real estate sector, to improve the attractiveness of real estate ads. The price of the house and the aesthetic characteristics are understood as the most dominant parameters that influence the purchase decision of individuals [22]. In addition, it is common that the decision to visit a property that fits the characteristics sought is marked by the attractiveness of its images.

In this work, we validate the aesthetic prediction system developed for the election of the online showcase in three portals and three different real estate agencies. In other words, we study the difference in impact between the ads that appear by default on the websites studied and the ads proposed by the system according to their aesthetics.

The work continues in the following way: in the Section 2 some works that present a proposal related to ours are briefly presented; in the Section 3 the potential of the aesthetic assessment system for its application in commercial tasks is explained; Section 4 presents the materials used and the methodology followed in the experimental phase; Section 5 and Section 6 present the results and discussion on them, respectively; and, finally, Section 7 gathers the conclusions of the work.

## 2. Related Work

In recent years there has been an increase in publications related to the study of digital images and their characteristics. However, there are few works of Computational Aesthetics or Artificial Intelligence applied to art and aesthetics that contemplate commercial applications or simply applications to the real world. The number of works is even lower if we talk about presenting validation methodologies of Computational Aesthetics systems. Next, we present and study two works that present a proposal for the use of aesthetics in electronic commerce, although focused on the fashion sector.

Chen and Allebach [23] designed a set of features for the inference of aesthetic quality in the context of online fashion shopping. They perform psychophysical experiments to build a database for the aesthetic evaluation of photos, specifically for photos from an online fashion shopping website. Later, Wang and Allebach [24] introduce new image features and apply the methodology of envelope feature selection with the “best-first” search algorithm to establish an optimal set of features that will produce the best predictive accuracy. With this, they manage to create an improved framework that achieves promising predictive accuracy.

Also related to the fashion sector, but with a focus closer to that which we propose, is the work of Yu et al. [25]. They propose to introduce the aesthetic information, which is very relevant to the user’s preference, in the garment recommendation systems. They used the Weka Machine Learning Package as a training and testing platform and trained a support vector regression model using 500 photos collected. 5-fold cross-validation was carried out to train the model parameters, and to ensure reliability. They achieve the best result with an RMSE value of 2.09 when using all feature sets. Their results show that their approach can capture the aesthetic preference of users and significantly outperform the more advanced recommendation methods.

## 3. Commercial Application

The aesthetic assessment system developed by our group has a strong potential in e-commerce since it allows ordering different commercial products according to the aesthetic value of their images.

The application of this system in electronic showcases allows products with the most attractive images to be shown in the first positions of the web. In a direct way, this should mean an increase in the number of clicks that visitors make on the web since they would find the products that are most interesting for them first. This premise will be validated with the experimental part of this work.

Indirectly, if we confirm that the previous premise is true, the ordering system according to the commercial attractiveness of the images also manages to (i) improve the user experience, (ii) improve the positioning of the company and (iii) increase the capture of new products.

## 4. Materials and Methods

### 4.1. Aesthetic Assessment System

Aesthetic image evaluation has usually been treated as a machine learning problem. The machine learning process is based on how the features of the image correspond to categories or aesthetic values. Due to the subjectivity of aesthetic evaluation, supervised learning methods are the most popular.

The aesthetic assessment system used in this work was created by our research group in recent years. It is based on a genetic algorithm whose objective is to create a model established in a multiple regression that maximizes the correlation (Spearman, Pearson or R-Square) by modifying the search space according to the values selected during the filtration.

**Figure 1:**
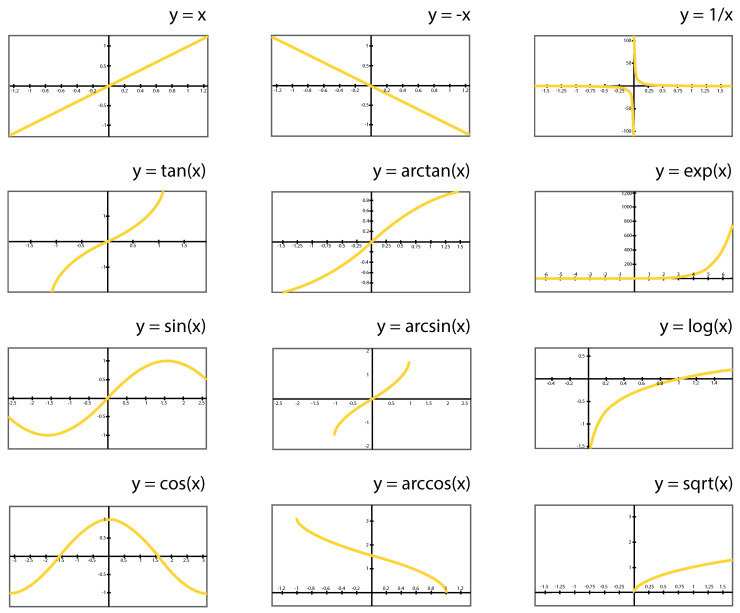
The 12 available sets of mathematical transformations were used to determine the evolutionary process [33].

The feature selection [26], feature transformation [27] and parameter selection [28] were made simultaneously to create an adjusted linear regression model using R-Squared as a measure of performance in the evolutionary process (instead of a measure of the error). The genetic algorithm uses mathematical operators to refactor input variables to find a suitable solution. The 12 available transformations in the evolutionary process used in this study are represented in the diagram (Figure 1). Any continuous mathematical function could have been used; however, this specific subset was selected based on previous experiences.

**Figure 2:**
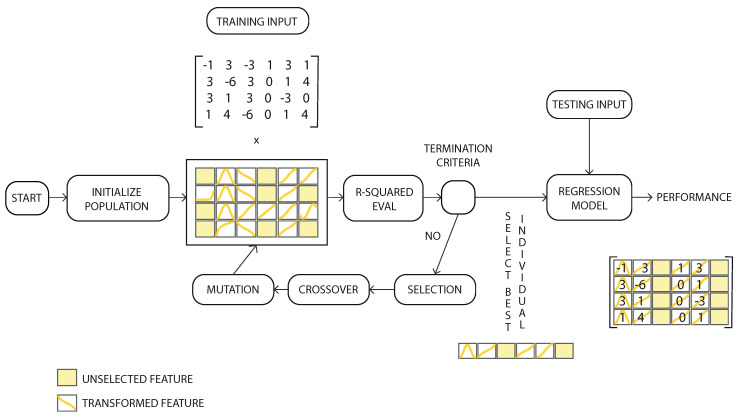
Workflow diagram of the hybrid genetic algorithm. The Feature Selection, Feature Transformation and Parameter Selection were made simultaneously to maximize the objective correlation function (R-Squared) [33].

The genetic algorithm attempts to calculate the best possible combination of selections and transformations for all the input features. The workflow of the hybrid genetic algorithm is shown on a flowchart (Figure 2). Through the evolutionary process, the best combinations of transformations in the input variables, which maximize the objective function, were determined.

As can be in the figure Figure 2 some of the features are not selected (not connected to any operator). Those unselected features are shown with a yellow colour. Other features (called in the figure: transformed feature) are connected to one of the functions shown in Figure 1. The output of the system is the sum of all the features after applying the corresponding operator.

For the best individuals of the population, the parameters with the best adaptation were selected (parameter selector) to create the final regression model. The termination criteria of the evolutionary process are connected to the performance of the current individuals of a population in relation to the average individual of the population. When the average difference is lower than the preset threshold value for a homogeneous population, the iterative search process comes to an end. For the cases in which the threshold value is not reached, a maximum number of iterations are used to finalize the process.

The metrics used by the system are metrics obtained through genetic programming [29], artificial neural networks (ANN) [30,31] and ad-hoc metrics [32].

This model was previously used successfully to predict the urban vertical growth in Tokio [33] and also in previous tasks of predicting the aesthetic value of images with transfer learning [32].

This system is protected by Industrial Secret by the company Artificial Intelligence Indestia S.L. (owner of PhotoILike.com).

### 4.2. Training Dataset

Currently, the system works with a dataset formed by images of the Spanish real estate park and with more than one million human evaluations, focused on tasks of increasing the commercial impact in the real estate sector. In order to create the dataset, thousand of existent Real Estate ads was randomly selected from Spanish Real Estate Portals (like Fotocasa, Idealista and Pisos.com). From each ad, it was selected a number of images. In 30% of the ads, all images were selected. In the remaining ones just 5 images were selected: The first and second ones of the ad, and three ones were randomly selected.

Every two months, new images are included in the dataset, following an internal mechanism to detect images that are wrongly predicted by the system.

Each of the images was being evaluated by several human beings using Amazon Mechanical Turk (AMT). The task of the human being at AMT is to determine the commercial attractiveness by scoring the image with a Likert scale (1: the least attractive–10: the most attractive). Some process was done in order to avoid random valuations.

After those processes, each image was associated with the average commercial attractiveness in Spain (as all the human beings in AMT are Spanish). An image with a high score means an image that probably will have a high number of visualizations if it is used as the primary image of a Real Estate ad.

### 4.3. Evaluation Dataset

The basic objective of this experiment is to check how many clicks the properties that appear by default on the real estate websites receive and how many clicks they would receive if those properties were the ones with the highest aesthetic value. For this purpose, a new dataset has been created with images of different real estate ads collected from the websites of three portals and three real estate agencies in Spain. The sources have been anonymized, so from now on they will be treated as Portal1, Portal2, Portal3, Agency1, Agency2 and Agency3.

The method of composition of the sets is presented below:

First, a search for properties is performed on each of the websites. In the case of the real estate portals, the location of the properties is limited to the city of A Coruña. In the case of real estate agencies, no search filter is applied, since they are companies that cover a smaller territory.

Then, a representative set of the properties is selected, which will be formed by the first results of the search. In the case of the portals, they will be <the first 250 and in the case of the real estate agencies, they will be the first 125. This difference is only due to the volume of properties handled by the platforms themselves. From this set, 20% of the properties that appear first in the search is selected, which will form the “original” set of each source. In the case of the portals, they will be the first 50 properties that appear in the search and in the case of the agencies, they will be the first 25.

The next step is to form the “aesthetic” set of each source. To do this, the first image of each ad in the initial representative set is evaluated (250 for portals and 125 for agencies) through the aesthetic assessment system described in Section 4.1. Once all the images have been evaluated, the 20% with the highest score is selected and the “aesthetic” set is constituted with them.

In this way, we have 12 different sets in total, 2 for each case study (for each portal and for each real estate agency): the “original” set with 20% of the images that appear first in the search and the “aesthetic” set with 20% of the images best evaluated by the aesthetic assessment system. Thus, each portal has an “original” set of 50 images and an “aesthetic” set of 50 images; and each agency has an “original” set of 25 images and an “aesthetic” set of 25 images.

**Figure 3:**
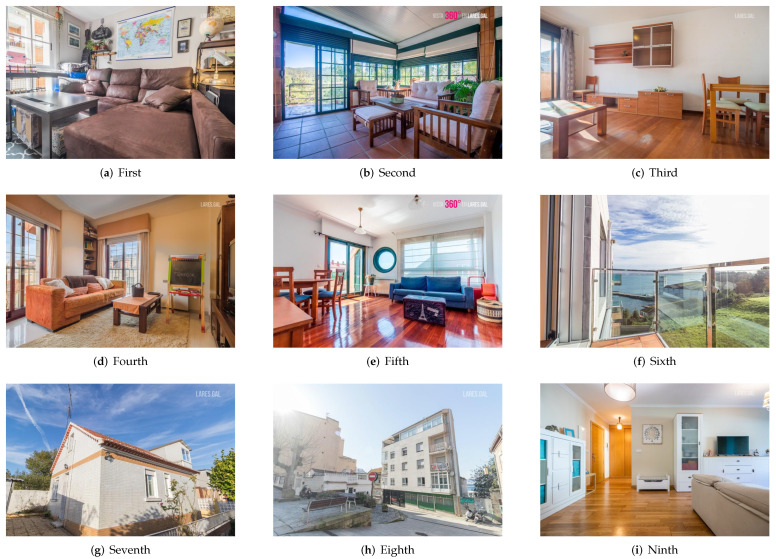
Images of the first nine ads from the “original” set of one of the agencies.

**Figure 4:**
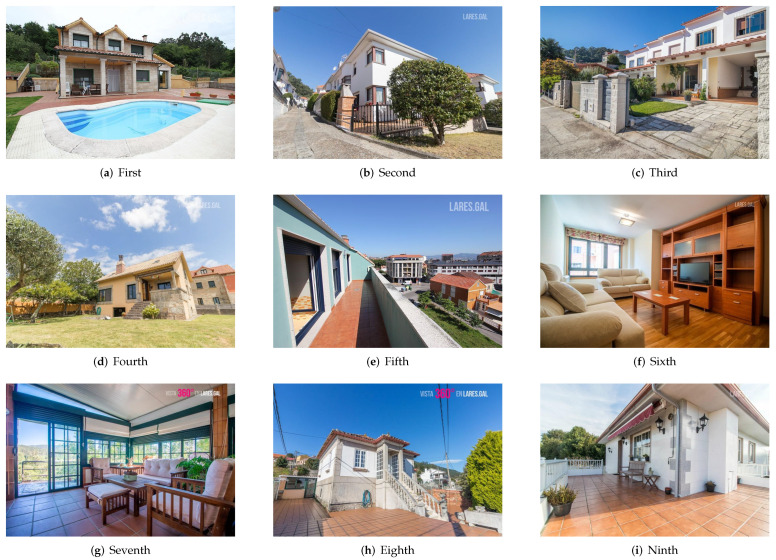
Images of the “aesthetic” set of one of the agencies proposed for the first nine positions.

In Figure 3 and Figure 4, a sample of the two sets from one of the agencies is presented. Figure 3 shows the nine images that appear first by default in the search and Figure 4 shows the nine with the highest aesthetic rating. Without going into detail, an important difference between the two sets can be observed: the images of the “original” set belong, for the most part, to rooms inside the houses and most of the “aesthetic” set is a photograph of the exterior.

When forming the image sets with the method described above, there is a possibility that some images are present in both sets, both in the “original” and in the “aesthetic”. For example, it may be the case that the image with the highest aesthetic value of one of the agencies is among the first 25 results of the search. Table 1 shows the number of images repeated in each case study and the percentage they represent of the total images from each source. In this case, we have decided to keep the repeated images in the experiment and count the number of votes they received for the result of both sets.

### 4.4. Sample and Procedure

As previously mentioned, this experiment aims to compare the number of clicks that the properties that appear by default in the search of each portal/real estate agency would receive and the number of clicks that those proposed by the evolutionary system of aesthetic evaluation would receive. To do this, a survey was conducted on a group of humans through the Amazon Mechanical Turk (AMT) tool.

In this experiment, the designed methodological process has great importance, since certain details could bias the response of the voters.

First, the images of the 12 sets described in the Section 4.3 are mixed at random, without taking into account the set to which they belong or the source of origin. Then, they are divided into groups of 10 images, also random, so that in each group there may be images from different sets and different sources. With these groups, the survey is designed in AMT.

**Figure 5:**
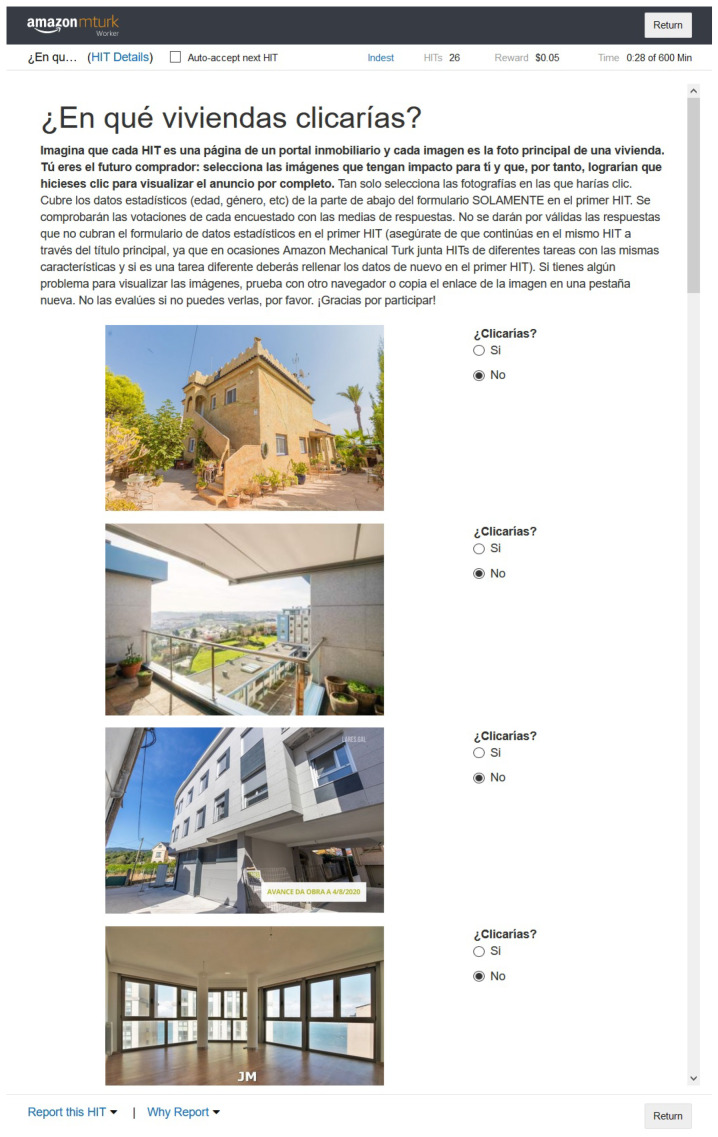
Example of the survey from the voter’s perspective.

The survey consists of a statement explaining to voters the purpose of the survey and then showing 10 images (only one group at a time is shown), each with two buttons next to it: one indicating that you would click on that image and another indicating the opposite. Figure 5 shows the survey as the individual would see it. Voters should check one option or the other, depending on whether they would click on that image if they saw it on a real estate website or not. There is no set number of images to mark, but each voter can indicate the number of images they would consider, regardless of the actual situation. By default, the answer marked on each image is “no”, so each voter must indicate the opposite if they think they are susceptible to receiving a click.

The full text of the statement seen by survey participants is presented below in English: “Imagine that each HIT is a page of a real estate portal and each image is the main picture of a house. You are the future buyer: select the images that have an impact on you and that, therefore, would make you click to view the entire ad. Just select the images you would click on. Cover the statistical data (age, gender, etc) at the bottom of the form ONLY in the first HIT. The votes of each respondent will be checked against the response averages. Answers that do not cover the statistical data form in the first HIT will not be considered valid (make sure you continue in the same HIT through the main title, since sometimes Amazon Mechanical Turk joins HITs of different tasks with the same characteristics and if it is a different task you will have to fill in the data again in the first HIT). If you have any problem viewing the images, try another browser or copy the image link to a new tab. Do not evaluate them if you can’t see them, please. Thank you for participating!”.

The survey design defined that each image should be evaluated by 50 people. However, AMT does not guarantee that all images will be evaluated by the same 50 people. Thus, a total of 61 people participated in our survey, although each image was evaluated by only 50 people as indicated. This is because AMT allows you to leave the survey at any time, so if a person evaluates only 30% of the images, the remaining 70% will be shown to another participant who has not gone through them.

The survey has the last part where some personal data related to the scope of the task is requested. In this case, only 81.6% of the participants answered this part of the survey, although it is a significant percentage and provides relevant data to the study. In Table 2 you can see the data obtained.

The group of voters in our experiment is composed of Spaniards between the ages of 18 and 48 with an average age of 35.6 years. These data are relevant in our study because the images to be evaluated belong to the Spanish real estate panorama, so they must be potential seekers of housing in Spain. Moreover, most of them (89.6%) have no studies or professional activity related to photography, design, architecture or similar activities that could bias their perception of the images. On the other hand, 93.9% live in a property they own or rent, so they have been and may continue to be potential home seekers.

## 5. Results

This section presents the results obtained in the survey carried out in AMT, with 50 evaluations per image. The results were divided into two tables, according to the origin of the data set. Table 3 shows the results for each of the three real estate portals that were taken to study. Table 4 shows the results obtained for the images of the real estate agencies.

The most outstanding result of this experiment is the fact that all the “aesthetic” sets surpass in clicks the “original” sets, both in portals and in real estate agencies.

In the case of the portals, as shown in Table 3, the “aesthetic” sets achieve an average total increase of 52.54%. The smallest increase is recorded in Portal1, with 32.71%, and the largest in Portal3, with 78.03%, passing through Portal2 with 53.58%. It should also be noted that the average number of votes per image in the “aesthetic” sets of the three portals is very similar, which suggests that the three portals have images of similar aesthetic value, only that by default they are ordered with a different criterion.

On the other hand, real estate agencies reveal much more varied results, as Table 4 shows. In this case, the “aesthetic” sets achieve an average total increase of 40.08%. The biggest and most outstanding increase in this group is that of Agency3, with 99.28%, followed by 33.12% for Agency1 and 23.27% for Agency2. The average number of votes per image in each of the “aesthetic” groups is also more varied, with 37.24 for Agency2, despite being the agency with the smallest increase in the number of clicks. This is probably because this agency’s images have a higher aesthetic value. In the case of Agency3, it can be deduced that, in addition to having photographs of lesser aesthetic value (it has the lowest average number of votes per image), the order that appears by default in the search is not attractive to users.

**Figure 6:**
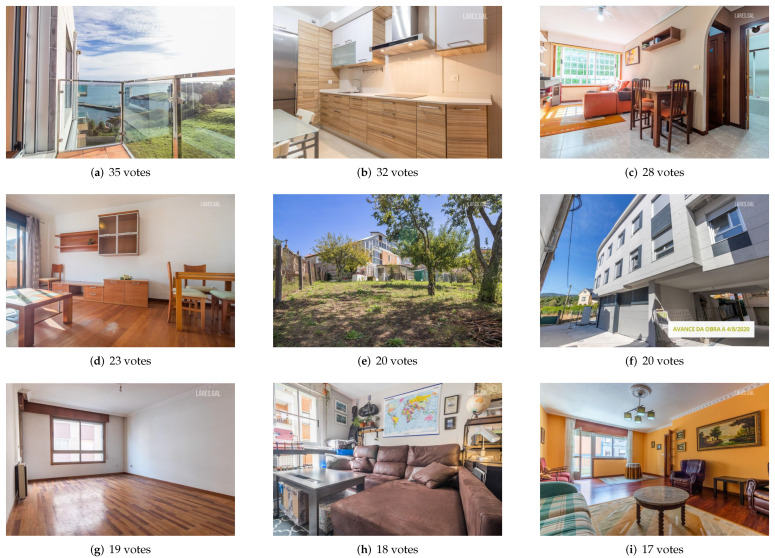
Images with more affirmative votes from the “original” set of one of the agencies.

**Figure 7:**
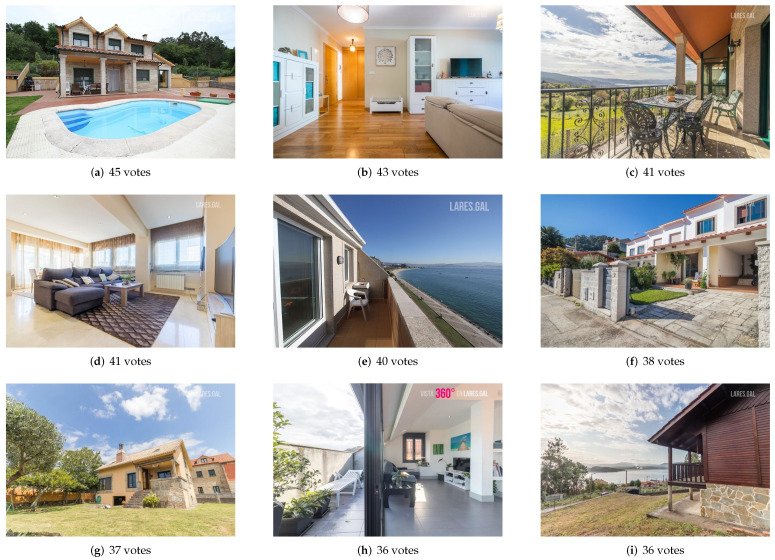
Images with more affirmative votes from the “aesthetic” set of one of the agencies.

Figure 6 and Figure 7 shows a sample of the images with more affirmative votes in both sets from one of the agencies. As can be seen and as already shown in the table of results, the images from the “aesthetics” set have received more votes than those from the “original” set, with the most voted being exceeded by 10.

### Statistical Analysis

Following the state of the art of Guyon [34] the Kolmogorov-Smirnov (K-S) test (goodness of fit test) was carried out, which serves to check whether a variable is normally distributed. The K-S test is more sensitive to values close to the median than to the extremes of the distribution, unlike the Anderson-Darling (A-D) test, which provides equal sensitivity at the extreme values [35,36]. In this case, it is not necessary to perform the A-D test. The results of the K-S test indicate whether or not to reject the null hypothesis that the data come from a normally distributed population [37]. Table 5 shows the statistical data for each of the sets: the original image set and the “aesthetic” one.

To perform the test we have a null hypothesis (H_0_) and an alternative hypothesis (H_1_):

**H_0_:**  *The sample data comes from a normally distributed population.*

**H_1_:**  *The sample data does not come from a normally distributed population.*

The higher the value of Kolmogorov’s D statistic, the less likely it is that your data will be normally distributed. The *p*-value quantifies this probability. Low probability indicates that the sample diverges from a normal distribution to a degree that is unlikely to arise by chance. That is, with a high Kolmogorov’s D value and a low *p*-value, the data is not normally distributed. The value of the test statistic for original images is 0.10467 and the value for “aesthetic” images is 0.10211. The *p*-value is 0.00292 and 0.004259 successively. Therefore, this provides good evidence that none of the datasets is normally distributed.

With these non-normality results, it is determined that we should use a non-parametric Wilcoxon signed-rank test [38,39,40]. The selection of this test comes from the state-of-the-art methodology, where it is recommended to use it in cases where the comparison is made only between two samples with some relation between the participants [41,42,43,44,45]. The studied sets meet the necessary conditions:

- The data must be dependent. Otherwise, we would have selected the Mann-Whitney U test [46,47].
- The data must be ordinal. This data must be able to be ordered from lowest to highest or vice versa.
- It is not necessary to assume that the samples are normally distributed or that they come from normal populations.
- Despite being considered the non-parametric equivalent of the t-test, the Wilcoxon signed-rank test works with medians, not means.
- Preferable to the t-test when there are outliers, there is no normality of the data or the size of the samples is small.

Again, for this test we have a new null hypothesis (H_0_) and an alternative (H_1_).

**H_0_:**  *There is no difference between one method or another.*

**H_1_:**  *The method in which the images are selected will be a key factor in whether or not the click occurs.*

The value obtained have been *W* = 27,392. The *p*-value is 1.978 $e^{-15}$. Therefore, the result is significant at *p* = 0.05 with an interval between −10.999984 and −6.999977.

After obtaining these results, therefore, we can affirm that there is a significant difference in the number of clicks depending on the selected dataset.

**Figure 8:**
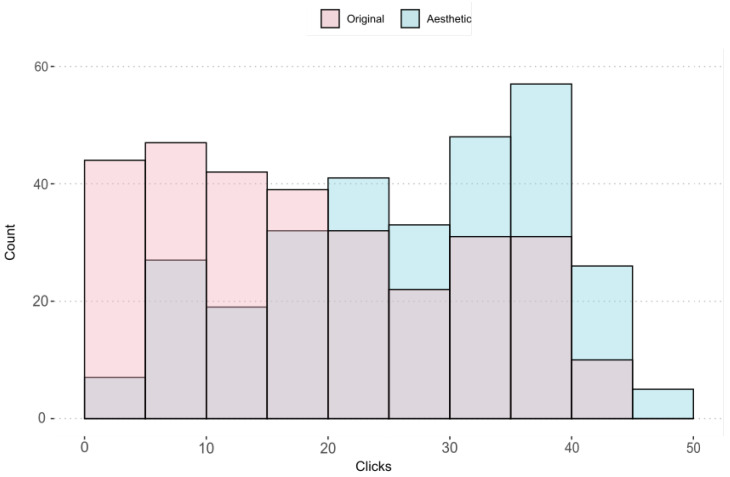
Chart showing the number of images within a range of clicks. Pink: click-through ratings on original ads. Blue: number of clicks on the images selected by the AI system.

We can see these results graphically in Figure 8. In this graph we observe the number of images for each rank of votes divided from 5 to 5. The columns in pink shows the number of clicks of the images selected in the original ads by the real estate agents themselves, while the columns in blue shows the clicks obtained on the images in our system. The values located to the left of the X-axis correspond to the least number of clicks, while the values located to the right correspond to the images with the highest number of clicks. Therefore, it is clear that in the total of images selected by advertisers, the number of clicks for each ad would not exceed the range 20–25 that can be observed on the X-axis. In contrast, the images selected by our system mainly cover the area with the most clicks on the graph.

## 6. Discussion

The results of the experiment were clear: the websites of the portals and real estate agencies would receive more clicks if they took into account the aesthetic value of their images when offering the search results. In this way, the stimulated emotional responses referred to by Deng and Poole [5] would be obtained and converted into more time surfing the website, exploring more properties or increasing the probability of visiting the property.

**Figure 9:**
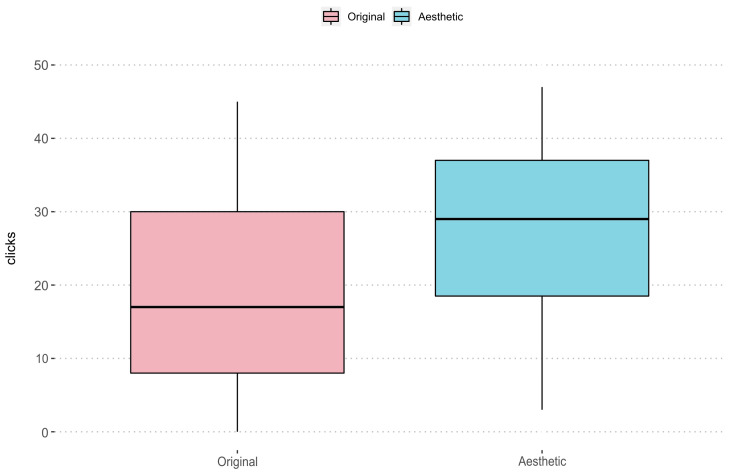
Boxplot distribution of both datasets. Pink: clicks of the “original” set. Blue: clicks of the “aesthetic” set.

From the statistical analysis carried out, we have been able to identify, as can be seen in Figure 9, that the selection of images based on the dataset has a significant influence on the number of clicks. If we look at the data from the “original” set (pink), we can see that 50% of the images have less than 17 clicks. It has positive asymmetry, but with a high dispersion range among its distribution. Regarding the “aesthetic” set (blue), the median is 29 clicks and, although it has a negative bias, its range of dispersion is smaller in this dataset. The *p*-value obtained with Wilcoxon allows us to say that the differences are highly statistically significant. Therefore, it can be identified that better results are obtained with the use of the “aesthetic” dataset.

There is a fact that deserves a special mention and it is the coincidence between the most voted image of the “aesthetic” set of one of the agencies (see (a) of the Figure 7) and the image proposed for the first position of that agency by the aesthetic assessment system (see (a) of the Figure 4). The system proposed for the first position the image that would receive the most clicks from potential buyers.

The portals and real estate agencies use different systems to order the properties by default in the search results. Some of them place in the first positions the most recent ads, while others use a relevance criterion that is not defined but that can take into account the existence of promoted ads (ads for which you pay extra money to occupy prominent positions). These criteria do not seem to influence significantly the results of the experiment.

What can be deduced from the results obtained is the aesthetic value of the photographs of the portals and agencies by the number of average votes received. In the case of the portals, they seem to have an average level of aesthetic value, although the results of Portal1 suggest that it has a slightly higher level. In the case of the agencies, Agency1 seems to have similar levels to those of Portal1, but the big differences appear in Agency2 and Agency3. The average number of votes received by these two suggest that Agency2 has photographs and advertisements of a higher aesthetic value than the rest of the agencies and portals, while Agency3 presents images with a notably lower quality.

Section 4.3 explained how 20% of the representative set was selected to form the two sets from each source. However, we have also analyzed how the results change when the percentage of images selected from the total set varies. Table 6 shows the average number of votes of the three real estate agencies studied, according to the percentage of images selected. In this case, in addition to the 20%, we have analyzed the variation with 10% and 40%. As you can see, in the original sets there are variations in the average votes when studying a different percentage of images. This is especially obvious in Agency1, where there is a significant increase in the average votes if we study only the first 10% of the set. Agency3 also shows better images in the 10% compared to 40%. However, Agency2 shows no significant variation between the different percentages.

The fact that some agencies show an improvement in the average number of clicks when a smaller percentage of the sample is studied, shows that there is an effort on the part of these agencies to show attractive properties in the first positions. We assume that this effort is because they consider that it improves the image of the agency and maximizes the number of clicks. However, the results obtained using the computer system are significantly better than those of the real estate agencies.

## 7. Conclusions and Future Directions

In this work, the real usefulness of a computerized aesthetic system has been demonstrated in the framework of a commercial application. Specifically, it has been demonstrated a significant increase in the impact of real estate ads by presenting images with greater aesthetic value.

For this, three portals and three different real estate agencies in Spain have been taken as case studies and a dataset has been created with images collected from their websites. From there, a validation method has been presented that can be adapted to different problems of computer aesthetics.

Significant results have been obtained, proposing an average increase in the number of clicks on the ads of 52.54% in the case of portals and 40.08% in the case of real estate agencies.

There are other ways to employ the Aesthetic assessment system. It can be employed to select the best image of each ad, so the ad gets more visits. There is actually a Real Estate portal in Spain (Kasaz) that employs the system to select the main image in all its ads. We will present the results of Proof of Concept that explore this use of the system in future papers.

Actually, the evaluation system, together with a tagging system that classifies the images in relation to the type of room (bedroom, kitchen, bathroom, exterior, facade) are used in a product that allows the clients to automatically order the images of an ad in less than 20 seconds. The automatic order will be both optimized to improve the conversion and organized in a way that all images of the same type of room will be together. The system can be tested for free with a limited number of images on the website PhotoILike.com.

About limitations, AI need always a dataset created with human evaluations. As the system showed in this paper is trained with Spanish Real Estate images evaluated originally with Spanish, it would not work as it is, with another country. It is needed to create a training dataset adapted to other countries or specific targets.

On another side, the system can be used in other domains (cars, second-hand ads, e-commerce) just by creating a human evaluated dataset.

In a more future application, it is possible to create a dataset for each user, so instead of having an average aesthetic value for culture, the system will predict the aesthetic taste of a particular user. We plan to explore this utility for e-commerce extreme personalising. In a simple example, when you look for tables in e-commerce like Ikea, you would find, in the first positions, those tables that are coherent with your aesthetic taste. Or if you have the permissions, the aesthetic taste of your couple or parent.

## Figures and Tables

**Table 1:** The number of images repeated in the “original” set and in the “aesthetic” set for each case study.

Set	Frequency	Proportion of Sample (%)
Portal1	6	12
Portal2	10	20
Portal3	9	18
Agency1	24	48
Agency2	16	32
Agency3	16	32

**Table 2:** Personal data provided by survey participants.

Item	Frequency	Proportion of Sample (%)
**Gender**		
Female	15	30.6
Male	34	69.4
**Age**		
25 and under	5	10.2
26–35	18	36.7
36–45	21	42.9
46 and over	5	10.2
**Income**		
600€ and under	4	8.2
601€–1200€	13	26.5
1201€–1600€	11	22.5
1601€–2000€	10	20.4
2001€–2500€	3	6.1
2501€–3000€	3	6.1
3001€ and over	5	10.2
**Related activities**		
Yes	5	10.4
No	43	89.6
**Habitat**		
Urban	37	75.5
Semi-urban	9	18.4
Rural	3	6.1
**Coexistence**		
Alone	5	10.2
With partner without children	16	32.7
With partner and children	17	34.7
With parents or other relatives	8	16.3
With other people (not relatives)	3	6.1
**Property**		
Rent	21	42.9
Ownership	25	51
Other	3	6.1
**Type of housing**		
House	9	18.4
Apartment	40	81.6

**Table 3:** The average number of votes per image received by each set, the increase for each portal and the total increase in portals. The maximum number of votes per image is 50.

	Portal1	Portal2	Portal3	Average
Original	21.40	17.45	16.02	18.29
Aesthetic	28.40	26.80	28.52	27.90
Increase	32.71%	53.58%	78.03%	52.54%

**Table 4:** The average number of votes per image that each group has received, the increase for each agency and the total increase in real estate agencies. The maximum number of votes per image is 50.

	Agency1	Agency2	Agency3	Average
Original	21.60	30.21	11.08	20.96
Aesthetic	28.76	37.24	22.08	29.36
Increase	33.15%	23.27%	99.28%	40.08%

**Table 5:** Statistical data for each of the sets: the original image set and the “aesthetic” one.

Value	Original	Aesthetic
Count	300	300
Mean	19.15771812	27.37288136
Median	17	29
Standard Deviation	12.28390138	11.44299433
Skewness	0.328208	−0.346432
Curtosis	−1.118261	−0.943309
Kolmogorov’s D	0.10467	0.10211
*p*-value	0.00292	0.004259

**Table 6:** The average number of votes per image received by each set of agencies according to the percentage of images analyzed.

	%	Agency1	Agency2	Agency3	Total
Original	10	24.00	29.27	13.16	66.43
Original	20	21.60	30.21	11.08	62.89
Original	40	19.97	29.97	10.42	60.63
Aesthetic	10	28.90	38.08	27.66	94.64
Aesthetic	20	28.76	37.24	22.08	88.08
Aesthetic	40	25.76	35.88	18.88	80.52

## Data Availability

Not applicable.
